# Characterizing Mental Health Treatment Utilization among Individuals Exposed to the 2001 World Trade Center Terrorist Attacks 14–15 Years Post-Disaster

**DOI:** 10.3390/ijerph16040626

**Published:** 2019-02-20

**Authors:** Melanie H. Jacobson, Christina Norman, Pablo Sadler, Lysa J. Petrsoric, Robert M. Brackbill

**Affiliations:** 1 World Trade Center Health Registry, Division of Epidemiology, New York City Department of Health and Mental Hygiene, New York, NY 10013, USA; Melanie.E.Jacobson@gmail.com (M.H.J.); lsilverstein@health.nyc.gov (L.J.P.); 2 Division of Mental Hygiene, New York City Department of Health and Mental Hygiene, Queens, NY 11101, USA; cnorman@health.nyc.gov (C.N.); psadler@health.nyc.gov (P.S.)

**Keywords:** counseling, post-disaster, psychotherapy, mental health treatment, treatment utilization

## Abstract

Following the World Trade Center (WTC) attacks in New York City (NYC) on 11 September 2001 (9/11), thousands in NYC experienced significant stress reactions and disorders, presenting an immediate need for counseling and treatment. While other studies documented post-9/11 mental health treatment utilization, none have data more than two years post-disaster. We used data from 35,629 enrollees of the WTC Health Registry, a longitudinal cohort study of those exposed to the WTC attacks, to examine predictors of counseling after 9/11, the types of practitioners seen, and the perceived helpfulness of therapy up to 15 years post-disaster. Among enrollees, 37.7% reported receiving counseling at some time after 9/11. Predictors of seeking counseling included race/ethnicity, age at 9/11, education level, exposure to the WTC attacks, other traumatic experiences, mental health symptomology, and pre-9/11 counseling. Whites and Hispanics, those who were children on 9/11, and those with high levels of exposure to the WTC attacks sought counseling soonest after 9/11. Among those who sought counseling, Blacks, Asians, and those with lower education and income were less likely to see mental health specialists and more likely to see general practitioners or religious advisors. Finally, among those who sought recent counseling, women, Blacks, those aged ≥65 years, and those with very high WTC exposures were more likely to rate their recent counseling as very helpful. This study used data up to 15 years post-disaster to document mental health treatment utilization patterns, trends, and disparities that have implications for future preparedness plans and needs assessments.

## 1. Introduction

The World Trade Center (WTC) terrorist attacks in New York City (NYC) on 11 September 2001 (9/11) resulted in thousands of casualties and, among the survivor population in NYC, a substantial mental health burden [1], primarily consisting of stress disorders [2,3]. Specifically, similar to the aftermath of other natural or human-made disasters, posttraumatic stress disorder (PTSD) was the most common mental health condition that resulted from the attacks [4]. This mental health burden translated into a need for crisis counseling and treatment [1]. In response, several mental health programs in New York City were established after 9/11, such as Project Liberty [5]. Project Liberty was a mental health screening and treatment program with a bilateral approach. The first tier was a general outreach program to communities affected by the attacks, providing free short-term counseling and education on coping methods for typical stress reactions. In addition, it included delivery of counseling to children in schools [6]. Secondly, individuals were screened for more severe and/or prolonged symptoms and then referred to specialized mental health treatment. Separately, mental health treatment was also offered through a program initially designed to monitor and treat those who participated in the rescue and recovery efforts [7]. Subsequently, treatment for mental health conditions was also made available to community members (i.e., those who were not involved in the rescue and recovery work) [8]. Together, these programs are collectively referred to as the WTC Health Program. Despite these established programs, it was consistently documented that receipt of these mental healthcare services was less than expected given the number of affected individuals and magnitude of the situation [9,10], which is common among populations who experience different types of mass trauma, such as natural disasters [11,12].

Several studies examined mental health treatment use after 9/11 and documented predictors of treatment receipt [9,13,14,15,16,17]. These studies reported that those with the greatest levels of exposure to the WTC attacks and those who had experienced peri-event panic attacks were the most likely to use mental health services after 9/11. Individuals who were Black and those without a regular doctor were less likely to use mental health services, including therapy and medication. However, these studies were conducted between six months and up to two years after the attacks, and data on longer-term or delayed utilization are sparse. Furthermore, studies generally lack specific details on the type of practitioner sought and the perceived degree of benefit of counseling.

The main objective of this study was to describe post-9/11 mental health treatment utilization, specifically counseling and therapy among individuals exposed to the WTC attacks up to 15 years post-disaster. Firstly, we examined predictors (e.g., demographics and exposure to the WTC attacks) of seeking counseling after 9/11, and then compared the characteristics of individuals who sought different types of practitioners for such counseling. Secondly, we assessed determinants of perceived benefit of recent counseling. Lastly, we evaluated the distributions of time to first counseling after 9/11 by several factors.

## 2. Materials and Methods

*Study Population*. The World Trade Center Health Registry (Registry) is a longitudinal cohort study of persons exposed to the WTC terrorist attacks on 9/11 [18]. Those who lived, worked, went to school, or were otherwise present in lower Manhattan on 9/11 and/or those who participated in the rescue and recovery efforts were eligible to enroll. The Registry was established in order to track the short and long-term potential health effects of 9/11.

The study design, eligibility, and enrollment methods of the Registry were previously described [18]. Briefly, in 2003–2004, 71,426 individuals who were exposed to the WTC attacks on 9/11 either as rescue and recovery workers or community members, were enrolled into the Registry and completed a baseline questionnaire (Wave 1). This was followed in subsequent years by Wave 2 (2006–2007), Wave 3 (2011–2012), and Wave 4 (2015–2016). The Institutional Review Boards of the Centers for Disease Control Prevention and the New York City Department of Health and Mental Hygiene approved the Registry protocol.

For this study, enrollees had to complete the Wave 4 questionnaire (*N* = 36,862), which included questions on their mental health treatment history, and were required to not be missing data on date of birth (*N* = 18) or missing a response to the question about whether the enrollee had at least one session of counseling or therapy after 9/11 (*N* = 1215). This yielded a final sample of 35,629.

*Mental health treatment*. On the Wave 4 survey, enrollees were asked about their mental healthcare-seeking behavior ever, after 9/11, and in the last 12 months. Firstly, enrollees were asked whether they had ever had a session of counseling or therapy lasting 30 minutes or longer and, if so, at what age the first session of counseling occurred, and whether any counseling was sought after 9/11. Among those who reported seeking care after 9/11, questions were asked about the conditions for which counseling was sought (e.g., depression, PTSD, anxiety, among others) and what types of practitioners were sought (e.g., psychologist, psychiatrist, social worker, clergy member, among others). Lastly, among those who reported seeking counseling in the last 12 months, questions were asked about medical indications for counseling, frequency of counseling, and perceived benefit. Whether any counseling was sought in relation to the events of 9/11 was not explicitly asked in the questionnaire.

*Explanatory variables*. Questionnaire data consisted of details on demographics and social factors, WTC-related exposures and experiences, health-related and care-seeking behaviors, and mental and physical health symptoms and conditions over time. Demographic information included sex, age, race and ethnicity, household income, and education. For this study focusing on mental health, exposure to the WTC attacks was operationalized using data on several traumatic experiences on and directly after 9/11 that were asked about on Waves 1 and 2. Based on work by Adams and Boscarino [19], Brackbill et al. derived a composite score consisting of 11 questions about traumatic experiences such as being in the North or South World Trade Center (WTC) towers at the time of the attack, witnessing three or more events (seeing planes hit the buildings, people fall or jump from buildings, people injured, or people running), being injured on 9/11; having a relative killed on 9/11, and being displaced from home due to 9/11 [20]. These items were summed (range = 0–11) and the score was then categorized as none/low (0–1 exposures), medium (2–3), high (4–5), and very high (≥6). In addition, another measure of “exposure” to the WTC attacks was the Registry eligibility group. Enrollees were categorized with regard to how they originally became eligible for the Registry: rescue and recovery workers, lower Manhattan residents or lower Manhattan area workers, passersby, or students. Because these groups were not mutually exclusive, those who met the criteria for more than one category were placed in the category considered to be more highly exposed to 9/11, such that rescue and recovery workers had greater levels of WTC exposure than residents, who had greater exposures than area workers, passersby, and students.

*Mental health measures*. PTSD symptoms were assessed at each wave (Waves 1–4) using the 9/11-specific PTSD Checklist (PCL)-17 [21,22,23]. The PCL is a self-administered questionnaire based on *Diagnostic and Statistical Manual of Mental Disorders* (DSM)-IV criteria [24] and its validity was established [25]. Total scores ≥44 were considered to be indicative of probable PTSD [21]. PTSD status was summarized across time as ever (scores ≥44 on at least one wave) or never (scores <44 on all waves). Persistent PTSD was defined as those who had scores ≥44 at all four waves.

Depressive symptoms were assessed using the Patient Health Questionnaire (PHQ)-8 [26] at Waves 3 and 4 only. This self-administered and validated instrument contains eight of the nine criteria that constitute the DSM-IV diagnosis of depressive disorders [24,27]. Scores ≥10 were considered to be indicative of moderate to severe depression [28].

Enrollees were also asked whether they had ever been diagnosed by a doctor or other medical professional with various mental health conditions, such as depression, PTSD, or anxiety.

*Statistical Analysis.* Firstly, we evaluated the distribution of personal characteristics, WTC exposures, and mental health symptomology by whether enrollees sought counseling at some time after 9/11. In order to identify predictors of seeking counseling, we then fit multivariable log binomial models to estimate associations between these factors and seeking counseling after 9/11. In these models, we did not include factors that required a doctor diagnosis (e.g., doctor-diagnosed depression), since these diagnoses could have been received through such counseling visits. Instead, we included PTSD and depression symptoms as measured by the PCL-17 and PHQ-8, respectively. We fit these models among everyone in the sample, and then conducted a sensitivity analysis restricting the sample to those who had a history of PTSD (Waves 1–4) or depression (Waves 3–4) via threshold PCL-17 and PHQ-8 scores, respectively, in order to examine those with the most theoretical clinical need for counseling. Next, among those who sought counseling, we then examined the types of practitioners sought, and the distributions of personal characteristics, WTC exposures, and mental health symptomology across practitioner type. Again, we did this in the total sample, and subsequently just among those with a history of PTSD or depression. Next, among those who had sought counseling in the last 12 months prior to completing the Wave 4 questionnaire, we explored the determinants of perceived helpfulness of therapy. We fit log binomial models estimating adjusted risk ratios (aRR) and 95% confidence intervals (CI) to identify predictors of perceiving recent counseling as “very helpful” compared with all other ratings (i.e., collapsing all other categories: somewhat, slightly, and not at all). Demographic characteristics, WTC-related exposures, mental health symptomology, and variables related to care-seeking were examined simultaneously in models. Finally, among those who did not seek counseling before 9/11, we assessed the distributions of time elapsed between 9/11 and first seeking counseling (i.e., time to first counseling after 9/11) by several covariates, such as demographics and WTC exposures using unadjusted Kaplan–Meier curves.

## 3. Results

**Figure 1:**
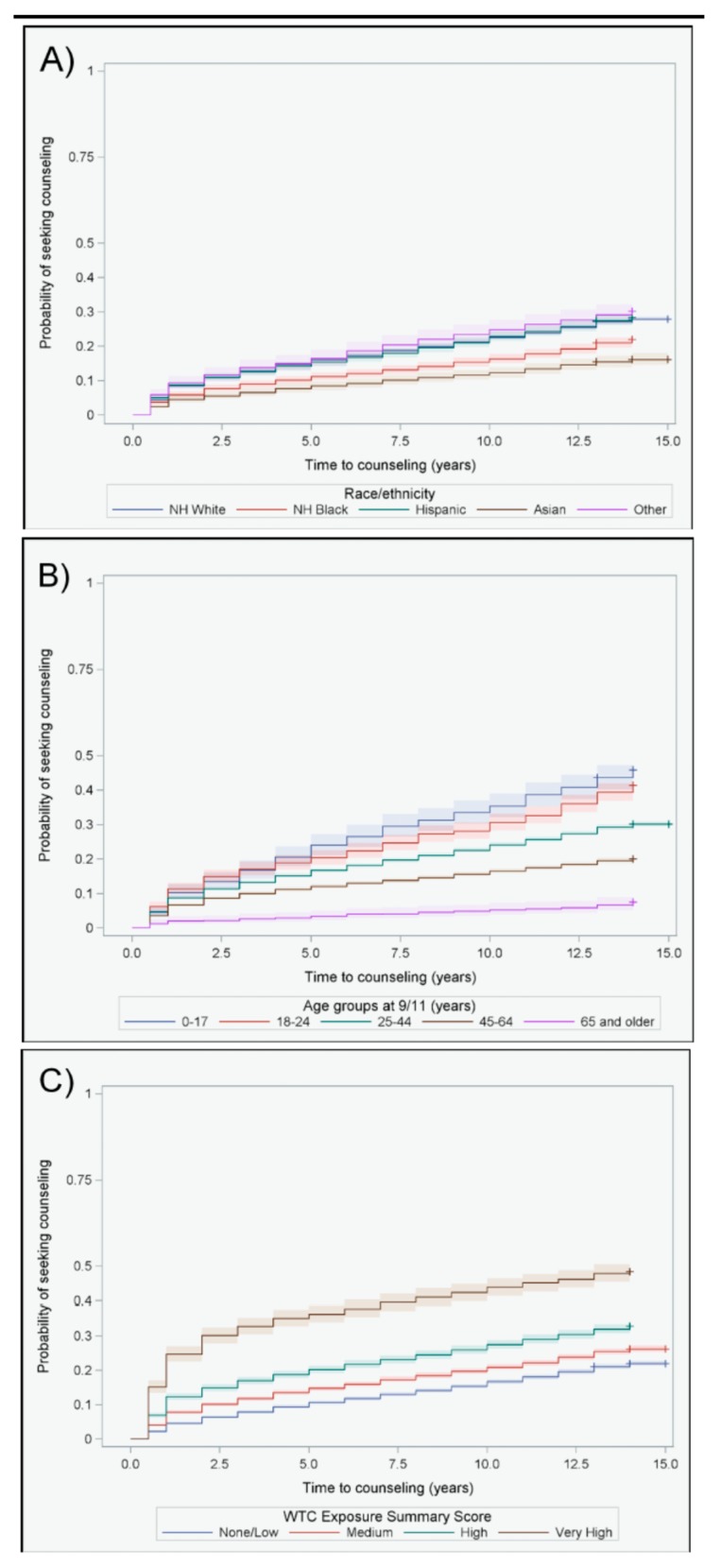
Unadjusted Kaplan–Meier curves and 95% confidence intervals of time to first counseling after 11 September 2001 (9/11) among those who had not sought counseling before 9/11 (*N* = 27,971), World Trade Center (WTC) Health Registry, 2003–2016: (**A**) by race/ethnicity; (**B**) by age at 9/11; (**C**) by WTC exposure summary score.

The study population was majority male (60.6%), White (70.0%), and had at least a college education (53.2%) (Table 1). There was approximately equal representation of rescue and recovery workers (46.3%) and community members (residents, area workers, passersby, and students; 53.7%) in the sample. Mental health conditions were common: 27.1% screened positive for PTSD on at least one Wave according to PCL-17 scores over time and 16.3% reported ever being diagnosed with PTSD by a medical professional. Likewise, although only 18.5% of individuals reported seeking counseling before 9/11, 37.7% reported counseling at some time after 9/11. Among those who sought treatment for the first time after 9/11, counseling was sought consistently throughout the 15 years after 9/11, with the largest increase observed in the first year after 9/11 (Figure 1).

Females were more likely to seek counseling compared with males (44.1% vs. 33.5%, although in adjusted models, sex was not a predictor of counseling (aRR = 1.01, 95% CI: 0.99, 1.03). While Blacks (aRR = 0.83, 95% CI: 0.76, 0.90) and Asians (aRR = 0.72, 95% CI: 0.63, 0.82) were less likely to seek counseling compared with Whites, Hispanics and those of other races were equally likely to seek counseling compared with Whites. This was also apparent in the examination of time to counseling (Figure 1A). Asians were the most likely to delay counseling after 9/11 (i.e., had the longest times to counseling) compared with those of other races (i.e., who had the shortest times). Age at 9/11 was a strong predictor of counseling. Those who were younger at 9/11, especially those aged 0–17 years, were the most likely to seek counseling compared with those aged 65 years and older (Table 1). This observation was consistent in the distributions of times to counseling (Figure 1B). Those who were children (0–17 years) at the time of 9/11 sought counseling sooner than those who were older. This relationship between age at 9/11 and time to counseling was monotonically positive such that, as age increased, the delay in seeking counseling also increased (i.e., longer times). Other demographic characteristics showed mixed associations with seeking counseling; while those with less than a high school education were slightly less likely to seek counseling compared with those with a graduate degree, income was not associated with seeking counseling (Table 1).

Measures of exposure to the WTC attacks, other post-9/11 traumatic experiences, and mental health symptomology were positively associated with seeking counseling. For example, those with very high scores of WTC exposure were more likely to seek counseling compared with those with none or low exposure (aRR = 1.10, 95% CI: 1.06, 1.14), and again this pattern was visible when times to counseling were considered (Figure 1C). Those who experienced the greatest number of traumatic exposures on 9/11 sought counseling soonest, such that there was a negative dose–response relationship between the WTC exposure summary score and time to counseling; the greater the number of exposures, the sooner counseling was sought (i.e., shorter times). Other traumatic experiences after 9/11 were similarly associated with an increased likelihood of seeking counseling (aRR = 1.08, 95% CI: 1.05, 1.12)

Although residents were the most likely to seek counseling (44.4%) compared with rescue and recovery workers (34.8%) or area workers and passersby (38.7%) in bivariate analyses, all were equally likely to seek counseling in adjusted models (Table 1). Those who had threshold PTSD symptoms at any wave (ever-PTSD), as well as those who had threshold depressive symptoms at Waves 3 and/or 4 (ever-depression), were more likely to seek counseling after 9/11 compared with those who did not meet symptom thresholds for each condition. Although not included in models, those who received diagnoses of depression, PTSD, and anxiety were more much more likely to have sought counseling compared with those without diagnoses. Finally, having sought counseling before 9/11 was a strong predictor of seeking counseling after 9/11 (aRR = 2.25, 95% CI: 2.16, 2.34).

When this analysis was conducted among those with PTSD or depression symptoms (*N* = 9391), results did not change (data not shown). Although the prevalence of having sought counseling after 9/11 was greater among those with PTSD or depression symptoms over time (57.1%) compared to the total (37.7%), the associations between enrollee characteristics and seeking counseling did not materially vary.

Among those who sought counseling after 9/11, psychiatrists (40.2%), psychologists (50.0%), and/or other mental health professionals (i.e., social worker, therapist, or counselor) (47.3%) were the most common types of practitioners sought (Table 2). Type of practitioners sought varied across most demographic characteristics, but did not vary by sex. Whites, Hispanics, and those of other races were more likely to see psychiatrists or psychologists compared with Blacks or Asians. However, Blacks and those of other races were more likely to seek counseling from nurses/occupational therapists or religious or spiritual advisors than Whites or Asians. Finally, Asians were the most likely to report seeking general practitioners for counseling compared with other race/ethnicity groups. In addition, although seeing a psychiatrist did not vary by education or income, these demographic characteristics were positively associated with seeing a psychologist such that those with the highest level of education or income were more likely to see a psychologist compared with those with lower levels. In contrast, those with lower education or income were more likely to see a general practitioner, nurse/occupational therapist, or religious advisor compared with those more educated and/or who earned more income.

Those who were children (aged 0–17 years) at 9/11 were more likely to seek counseling from mental health specialists (e.g., psychologists, psychiatrists, or other mental health professionals) compared with those who were older. However, older individuals, especially those 65 years and older, were more likely to seek general practitioners and nurses for counseling compared with those of younger age groups, whereas middle aged individuals were the most likely to see religious advisors. Rescue and recovery workers, residents, and area workers/passersby generally saw different practitioners with similar frequencies, although residents were the least likely to seek counseling from religious or spiritual advisors (9.0%) compared with rescue and recovery workers (18.5%) or area workers/passersby (15.2%).

Those with high WTC exposures were more likely to see all types of practitioners compared with those with lower exposures. The most common practitioners sought overall were psychologists; 56.2% of those with high WTC exposures reported seeing a psychologist for counseling. Similarly, those with threshold PTSD or depression symptoms were more likely to seek counseling from all types of practitioners compared with those without symptoms, although these differences were smallest for seeking counseling from other mental health professionals. Specifically, 47.4% of those who never had threshold PTSD symptoms reported seeking counseling from other mental health professionals compared with 50.2% of those who did have threshold PTSD symptoms. This pattern was similar across those who reported receiving a formal diagnosis of depression, PTSD, or anxiety compared with those who did not. There was also little difference between the types of practitioners sought comparing those who had sought counseling before 9/11 and those who had not. Finally, the conditions for which individuals sought counseling affected the type of practitioner they sought. Specifically, more of those who sought counseling for alcohol or drug problems saw all types of practitioners other than psychiatrists or psychologists, such as general practitioners, nurses/occupational therapists, and religious advisors, compared to those who sought counseling for other conditions. Overall, these trends did not change when the sample was restricted to those who experienced PTSD or depression symptoms over time (data not shown).

Lastly, among those who reported receipt of counseling within the last 12 months (*N* = 5429), the vast majority (79.3%) reported that the counseling was at least somewhat helpful (Table 3). Women (vs. men), Blacks (vs. all other race/ethnicities), and those aged ≥65 years at 9/11 (vs. all younger age groups) were more likely to report that therapy was very helpful. In bivariate analysis, similar distributions of perceived helpfulness were observed across eligibility groups and WTC exposure levels; however, in adjusted models, those with very high WTC exposure scores (aRR = 1.21, 95% CI: 1.07, 1.36) were more likely to rate their recent counseling as very helpful compared with those with none or low levels of WTC exposures.

Those with persistent PTSD were less likely to rate their recent counseling as very helpful (aRR = 0.68, 95% CI: 0.58, 0.81), and more likely to report that it was “not at all” (5.4%) or slightly” (21.8%) helpful compared with those who never had PTSD (2.2% and 12.3%, respectively). Those who had non-persistent PTSD (i.e., intermittent, delayed, or recovered) were also less likely to rate their recent counseling as very helpful compared with those who never had PTSD (aRR = 0.76, 95% CI: 0.68, 0.83). Although similar frequencies of helpfulness were observed across the conditions for which people received counseling, in adjusted models, those who received counseling for depression (vs. all other indications) were less likely to rate their counseling as very helpful, whereas those who received counseling for PTSD (vs. all other indications) were more likely to rate their counseling as very helpful.

The frequency of counseling was positively associated with perceived helpfulness such that those who sought counseling more often reported it to be “very helpful” compared with those who went less often. Similarly, those who had received therapy prior to the last 12 months were more likely to report that their current counseling was very helpful compared with those who had never before received therapy (aRR = 1.34, 95% CI: 1.14, 1.57). Those who received medication for a mental health problem in the last 12 months were slightly less likely to report that their counseling was very helpful (aRR = 0.91, 95% CI: 0.83, 0.99) compared with those who did not receive medication, although this difference was small (38.8% vs. 44.4%, respectively) and not consistent among those who reported that it was somewhat helpful (39.4% vs. 36.4%, respectively). Finally, those who reported unmet mental healthcare needs in the last 12 months and previously were less likely to report that their therapy was very helpful compared to those who did not have unmet mental healthcare needs.

## 4. Discussion

In a large cohort of individuals exposed to the trauma of the WTC disaster, this study documented mental health treatment utilization up to 15 years after 9/11. Approximately one-third of Registry enrollees sought counseling at some time after 9/11. Counseling was sought consistently over time after 9/11 up to 15 years after, although the largest increase was observed within the first year after the disaster. Predictors of seeking counseling included race/ethnicity, age at 9/11, education level attained, exposure to the WTC attacks, other post-9/11 traumatic experiences, mental health symptomology, and pre-9/11 counseling. Whites, Hispanics, and those of other races, those who were children at the time of 9/11, and those with high levels of exposure to the WTC attacks sought counseling soonest after 9/11. Among those who sought counseling, several trends were identified across types of practitioners seen. For example, Blacks, Asians, and those with lower education and income were less likely to seek counseling from mental health specialists (e.g., psychologists) and more likely to seek counseling from general practitioners such as family doctors or religious advisors compared with their White and more highly educated counterparts. Finally, among those who sought recent counseling, most enrollees perceived their counseling to have been at least somewhat helpful. Women, Blacks, those aged ≥65 years, and those with very high WTC exposures were more likely to rate their recent counseling as very helpful. These predictors of counseling and time to counseling after 9/11, types of practitioners seen, and perceived helpfulness of recent counseling did not vary when the population was restricted to those with significant PTSD or depression symptoms.

One of the strongest predictors of seeking counseling after 9/11 was having sought counseling before 9/11. Another study among 9/11-exposed individuals reported that new uptake of mental healthcare was rare after 9/11 among those who were not already receiving care beforehand [9]. Although this study was conducted only six months after 9/11, whereas ours was conducted 15 years after 9/11, results were very similar. Stuber et al. reported that, among those who were already receiving mental health services before 9/11, 82.7% sought mental healthcare after 9/11, which is comparable to the 75.9% in our study. In addition, we found that those who sought counseling more often, as well as those who were previously connected to care, were more likely to rate their recent counseling as very helpful compared to those who went less frequently or had not sought care prior. This may indicate that those with established provider–patient relationships, or those who are accustomed to seeking counseling fare better than those just starting out [29]. Alternatively, those already connected to care and those who go often may be experiencing more symptom abatement and, thus, satisfaction compared with those who may have intermittent care that may not be satisfying their needs [30]. This correlation between symptom abatement and reports of perceived benefit is consistent with our observation that persistent PTSD was associated with a reduced degree of perceived helpfulness of recent therapy compared with those who never had PTSD.

Another strong predictor of seeking counseling after 9/11 was younger age at the time of the event. Specifically, those who were children at the time of 9/11 were the most likely to seek mental health treatment and sought treatment more quickly compared with those of older ages at 9/11. This may reflect counseling programs that were provided in schools [31,32], as well as parents worrying about children’s potential needs after the disaster [6,33]. Given the vulnerable life stage of children who were exposed to 9/11 and the resulting psychological consequences that are thought to be of greater severity in this age group [34,35], this relatively greater degree of uptake is reassuring. However, in our study, the absolute proportions show a different perspective, with just over half of those aged 0–17 years at 9/11 having sought counseling at some time after 9/11. This represents an underutilization of the services that were available, especially in the aftermath of the events, both in this age group and in the overall population. In addition, we found that these young individuals were the least likely to rate any recent counseling as very helpful compared with older age groups. However, it should be noted that, for the majority of those who sought counseling in the 12 months before survey administration (i.e., recent counseling), the indication was likely not related to 9/11. However, we did not explicitly ask whether the counseling sought was to address 9/11-related trauma.

Consistent with other studies in post-disaster settings [12], this study documented an underutilization in counseling and mental health treatment. Despite several public health programs devoted to mental health, only about one-third of enrollees reported seeking counseling at some time in the 15 years after 9/11. This is similar to what was observed in the aftermath of Hurricane Katrina, although studied time frames were shorter [36]; however, even so, after initiation, drop-outs in treatment were common. In addition, we observed significant delays in seeking counseling across several strata. These types of delays in seeking treatment are common [37,38]. One major driver of utilization of care and delay in seeking it that was consistently documented is stigma [39,40]. Although we did not measure this, it was shown that certain populations are differentially more likely to be affected by stigma, including Blacks and Asians, males, and young people. Future studies in 9/11-exposed populations should explore race/ethnicity-specific barriers to care, including perceived stigma.

Another relevant issue for mental healthcare delivery in post-disaster settings is that natural reactions to disasters change over time [41]. The course of reactions is generally referred to as “threat” or “impact” (i.e., immediate), short-term, and long-term. These different phases present the need for different types of mental healthcare support. For example, in the aftermath of the Hanshin Awaji earthquake in 1995, depressive symptoms did not manifest in most of the affected population until weeks to months after the event [42]. This presents a very different need than the shock- and grief-related reactions that are more common in the “impact” phase [41]. Specifically, we reported a racial/ethnic disparity in receipt of counseling. Blacks and particularly Asians were the least likely to seek counseling after 9/11 compared with other racial and ethnic groups. It was repeatedly noted in the literature that Asian Americans are less likely to seek mental health treatment compared with other racial and ethnic groups, especially non-Hispanic Whites [43,44,45,46,47,48,49]. This is attributed to language barriers, deficiencies in cultural competence in the delivery of care, general lack of awareness of such service availability, and cultural differences in the conception of mental illness itself. These data have implications for improving the accessibility of culturally competent mental health services for Blacks and Asians.

This study benefited from several strengths. Firstly, this study provided long-term information on mental health treatment utilization after 9/11. Most published studies to date reported on mental health treatment utilization up to a maximum of two years post-disaster [15,17], whereas this study had data up to 15 years after 9/11. This is an important addition to the literature because we were able to document the degree of delays in treatment in a trauma-exposed population. However, it should be noted that, due to this long follow-up time, our examination of mental health treatment likely included visits related to 9/11, as well as utilization unrelated to the events of 9/11, and we were unable to discern between the two. Another attribute was that, although several studies were published on the topic of post-9/11 mental health service utilization, to our knowledge, this is the first that collected data on the type of practitioner sought. This type of information is important because we identified trends and disparities in care-seeking behavior across various demographics. These findings may inform future disaster response plans with regard to establishing more equitable care to all those potentially affected.

However, we also note this study’s limitations. Firstly, the Wave 4 survey asked about age at first counseling, irrespective of 9/11. Thus, our analysis of time to counseling after 9/11 was limited to those who sought counseling for the first time after 9/11. This limited our ability to examine the determinants of time to counseling after 9/11 among those who had sought treatment before 9/11 as well. Secondly, despite the information we had over time on several mental health conditions and symptoms, we were not able to assess whether mental health treatment was associated with improvement in symptoms because we did not ask detailed questions over time on treatment initiation and continuation or types of therapy and specific medication use and duration of use. In order to answer this question, in addition to longitudinal data on mental health symptomology, an in-depth study would be necessary that asked about specific treatment modes, practitioner characteristics, duration of treatments, use of medications over time, and more. Thirdly, although we asked about the type of practitioner sought for counseling, it is possible that enrollees were not able to reliably report the specific type, especially more subtle distinctions such as psychologists vs. psychiatrists. However, we were able to observe differences and disparities across different providers. Fourthly, we did not collect data on specific mental health or neurodevelopmental conditions before 9/11. Having this data would have allowed us to explore whether these disorders conferred an additional risk of developing PTSD after 9/11 [50], and perhaps an increased likelihood of seeking treatment. Lastly, our study was limited to Registry enrollees who completed the Wave 4 survey, which was administered in 2015–2016. These enrollees represented just over half (51.6%) of those originally enrolled in 2003–2004. Therefore, selection bias is a concern due to the potential for selective participation across several strata. However, previous investigation of this issue showed that, although those with PTSD symptoms were slightly less likely to continue to participate in Registry surveys than those without, the degree of exposure to the WTC attacks was not associated with participation over time [51]. Furthermore, the Registry was not able to enroll all WTC-exposed individuals, which was estimated to be over 400,000, of which the Registry recruited more than 71,000 (17.4% enrollment rate) [52]. The enrollment rate was highest among rescue and recovery workers (33.5%) and lowest among passersby (12.0%). Unfortunately, however, we do not have information on those who did not enroll.

## 5. Conclusions

The WTC terrorist attacks exposed thousands, if not millions, of individuals in NYC to trauma, resulting in a significant mental health burden and subsequent need for services. Overall, approximately one-third of WTC-exposed individuals sought counseling up to 15 years post-9/11, which represents an underutilization that is consistent with other post-disaster literature. Those who were White or Hispanic, children at the time of 9/11, and had high levels of exposure to the WTC attacks were the most likely to seek counseling after 9/11 and had the shortest waiting times to seeking counseling. Among those who sought counseling, there was heterogeneity across several demographic strata in the types of practitioners seen, such as Blacks and Asians being less likely to seek counseling from mental health specialists compared with Whites. These results highlight the need for tailoring outreach to specific demographic subgroups in post-disaster settings. This study used data up to 15 years post-disaster to document mental health treatment utilization patterns, trends, and disparities that have implications for future preparedness plans and needs assessments.

## Figures and Tables

**Table 1:** Distributions of personal characteristics, World Trade Center (WTC) exposures, and mental health symptomology by seeking counseling post 11 September 2001 (9/11) and adjusted risk ratios (aRR) and 95% Confidence Intervals (CI). GED—General Education Development; PTSD—posttraumatic stress disorder.

	Total (*N* = 35,629)		Sought Counseling (*N* = 13,435, 37.7%)		No Counseling (*N* = 22,194, 62.3%)		aRR ^a^	95% CI	
	*N*	%	*N*	%	*N*	%			
Sex									
Men	21586	60.6	7240	33.5	14346	66.5	1.00	Reference	
Women	14043	39.4	6195	44.1	7848	55.9	1.01	0.99	1.03
Race/Ethnicity									
White	24939	70.0	9857	39.5	15082	60.5	1.00	Reference	
Black	3416	9.6	1045	30.6	2371	69.4	0.83	0.76	0.90
Hispanic	4046	11.4	1550	38.3	2496	61.7	0.98	0.94	1.02
Asian	2002	5.6	470	23.5	1532	76.5	0.72	0.63	0.82
Other race	1226	3.4	513	41.8	713	58.2	1.00	0.99	1.02
Education at Wave 1									
≤High school/GED	7857	22.2	2619	33.3	5238	66.7	0.94	0.90	0.99
Some college	8704	24.6	3138	36.1	5566	63.9	0.99	0.96	1.02
College	11423	32.3	4470	39.1	6953	60.9	0.99	0.98	1.00
Graduate degree	7376	20.9	3115	42.2	4261	57.8	1.00	Reference	
Income at Wave 1									
<$50,000	8876	27.6	3632	40.9	5244	59.1	1.00	0.98	1.01
≥$50,000 to <$150,000	19249	59.8	6974	36.2	12275	63.8	0.99	0.98	1.00
≥$150,000	4050	12.6	1623	40.1	2427	59.9	1.00	Reference	
Age at 9/11 (years)									
0–17	852	2.4	438	51.4	414	48.6	2.44	1.86	3.20
18–24	1720	4.8	845	49.1	875	50.9	2.42	1.84	3.17
25–44	18212	51.1	7442	40.9	10770	59.1	2.37	1.81	3.11
45–64	14010	39.3	4551	32.5	9459	67.5	2.08	1.58	2.73
≥65	835	2.3	159	19.0	676	81.0	1.00	Reference	
Eligibility group									
Rescue/recovery worker	16480	46.3	5732	34.8	10748	65.2	1.00	0.98	1.01
Lower Manhattan resident	5103	14.3	2266	44.4	2837	55.6	0.99	0.95	1.02
Lower Manhattan area worker/passerby	14046	39.4	5437	38.7	8609	61.3	1.00	Reference	
WTC exposure score									
None/low	15620	43.8	5083	32.5	10537	67.5	1.00	Reference	
Medium	11923	33.5	4518	37.9	7405	62.1	1.08	1.04	1.12
High	6203	17.4	2743	44.2	3460	55.8	1.09	1.04	1.13
Very high	1883	5.3	1091	57.9	792	42.1	1.10	1.06	1.14
Traumatic experiences after 9/11 ^b^									
No	21859	61.4	6700	30.7	15159	69.3	1.00	Reference	
Yes	13770	38.6	6735	48.9	7035	51.1	1.08	1.05	1.12
Ever-PTSD ^c^									
No	17572	72.9	5156	29.3	12416	70.7	1.00	Reference	
Yes	6543	27.1	3726	56.9	2817	43.1	1.27	1.20	1.33
Ever-Depression ^d^									
No	22209	77.5	6691	30.1	15518	69.9	1.00	Reference	
Yes	6451	22.5	3973	61.6	2478	38.4	1.17	1.11	1.23
Counseling before 9/11									
No	27971	81.5	7460	26.7	20511	73.3	1.00	Reference	
Yes	6340	18.5	4810	75.9	1530	24.1	2.25	2.16	2.34
Doctor-diagnosed depression (ever)									
No	28099	78.9	7366	26.2	20733	73.8	-	-	
Yes	7530	21.1	6069	80.6	1461	19.4	-	-	
Doctor-diagnosed PTSD (ever)									
No	29816	83.7	8490	28.5	21326	71.5	-	-	
Yes	5813	16.3	4945	85.1	868	14.9	-	-	
Doctor-diagnosed anxiety (ever)									
No	30950	86.9	9695	31.3	21255	68.7	-	-	
Yes	4679	13.1	3740	79.9	939	20.1	-	-	

^a^ Risk ratio represents the comparison of seeking counseling vs. not seeking counseling (reference). ^b^ Traumatic experiences were defined as one or more of the following: experiencing a serious accident (e.g., in a car or a fall), an intentional attack with or without a weapon, forceful unwanted sexual contact, and serious family or work problems. ^c^ As measured by a score of ≥44 on the 9/11-specific PTSD Checklist (PCL)-17 on at least one wave (Waves 1–4). ^d^ As measured by a score of ≥10 on Patient Health Questionnaire (PHQ)-8 on at least one wave (Waves 3–4).

**Table 2:** Distributions of personal characteristics, WTC exposures, and mental health symptomology among those that sought counseling post-9/11 by type of practitioner.

	Total (N = 13,435)		Psychiatrist (*N* = 5399, 40.2%) ^a^		Psychologist (*N* = 6720, 50.0%) ^a^		Other Mental Health Professional (*N* = 6360, 47.3%) ^a,b^		General Practitioner/Doctor (*N* = 3082, 22.9%) ^a^		Nurse/ Occupational Therapist (*N* = 726, 5.4%) ^a^		Religious or Spiritual Advisor (*N* = 2092, 15.6%) ^a^	
	*N*	%	*N*	%	*N*	%	*N*	%	*N*	%	*N*	%	*N*	%
Sex														
Men	7240	53.9	3009	41.6	3591	49.6	3376	46.6	1650	22.8	404	5.6	1155	16.0
Women	6195	46.1	2390	38.6	3129	50.5	2984	48.2	1432	23.1	322	5.2	937	15.1
Race/Ethnicity														
White	9857	73.4	3998	40.6	5140	52.1	4759	48.3	2118	21.5	432	4.4	1362	13.8
Black	1045	7.8	369	35.3	419	40.1	458	43.8	272	26.0	95	9.1	240	23.0
Hispanic	1550	11.5	649	41.9	703	45.4	713	46.0	408	26.3	123	7.9	308	19.9
Asian	470	3.5	142	30.2	178	37.9	186	39.6	143	30.4	32	6.8	48	10.2
Other race	513	3.8	241	47.0	280	54.6	244	47.6	141	27.5	44	8.6	134	26.1
Education at Wave 1														
≤High school/GED	2619	19.6	1126	43.0	1156	44.1	1115	42.6	726	27.7	209	8.0	419	16.0
Some college	3138	23.5	1293	41.2	1513	48.2	1578	50.3	805	25.7	197	6.3	575	18.3
College	4470	33.5	1729	38.7	2328	52.1	2142	47.9	936	20.9	203	4.5	650	14.5
Graduate degree	3115	23.4	1215	39.0	1686	54.1	1481	47.5	585	18.8	109	3.5	420	13.5
Income at Wave 1														
<$50,000	3632	29.7	1521	41.9	1718	47.3	1778	49.0	1007	27.7	300	8.3	676	18.6
≥$50,000 to <$150,000	6974	57.0	2729	39.1	3523	50.5	3399	48.7	1548	22.2	327	4.7	1064	15.3
≥$150,000	1623	13.3	659	40.6	881	54.3	651	40.1	282	17.4	38	2.3	185	11.4
Age at 9/11 (years)														
0–17	438	3.3	203	46.3	258	58.9	232	53.1	50	11.4	22	5.0	40	9.2
18–24	845	6.3	331	39.2	468	55.4	442	52.3	157	18.6	32	3.8	114	13.5
25–44	7442	55.4	3033	40.8	3787	50.9	3726	50.1	1686	22.7	384	5.2	1270	17.1
45–64	4551	33.9	1791	39.4	2168	47.6	1922	42.2	1141	25.1	268	5.9	656	14.4
≥65	159	1.2	41	25.8	39	24.5	38	23.9	48	30.2	20	12.6	12	7.5
Eligibility group														
Rescue/recovery worker	5732	42.7	2287	39.9	2811	49.0	2910	50.8	1361	23.7	354	6.2	1059	18.5
Lower Manhattan resident	2266	16.9	956	42.2	1217	53.7	978	43.2	457	20.2	120	5.3	205	9.0
Lower Manhattan area worker/passerby	5437	40.5	2156	39.7	2692	49.5	2472	45.5	1264	23.2	252	4.6	828	15.2
WTC exposure score														
None/low	5083	37.8	1901	37.4	2416	47.5	2364	46.5	1092	21.5	238	4.7	769	15.1
Medium	4518	33.6	1797	39.8	2227	49.3	2098	46.4	918	20.3	213	4.7	585	12.9
High	2743	20.4	1141	41.6	1464	53.4	1320	48.1	705	25.7	161	5.9	487	17.8
Very high	1091	8.1	560	51.3	613	56.2	578	53.0	367	33.6	114	10.4	251	23.0
Ever-PTSD ^c^														
No	5156	58.1	1544	29.9	2361	45.8	2445	47.4	709	13.8	132	2.6	561	10.9
Yes	3726	42.0	1962	52.7	2122	57.0	1872	50.2	1201	32.2	288	7.7	744	20.0
Ever-Depression ^d^														
No	6691	62.7	2018	30.2	3105	46.4	3103	46.4	1011	15.1	195	2.9	821	12.3
Yes	3973	37.3	2241	56.4	2290	57.6	2033	51.2	1359	34.2	342	8.6	803	20.2
Counseling before 9/11														
No	7460	60.8	3014	40.4	3811	51.1	3576	47.9	1624	21.8	414	5.5	1137	15.2
Yes	4810	39.2	2105	43.8	2612	54.3	2476	51.5	1086	22.6	233	4.8	760	15.8
Doctor-diagnosed depression (ever)														
No	7366	54.8	1642	22.3	3098	42.1	3245	44.1	1088	14.8	240	3.3	973	13.2
Yes	6069	45.2	3757	61.9	3622	59.7	3115	51.3	1994	32.9	486	8.0	1119	18.4
Doctor-diagnosed PTSD (ever)														
No	8490	63.2	2568	30.2	3690	43.5	3645	42.9	1511	17.8	304	3.6	1065	12.5
Yes	4945	36.8	2831	57.2	3030	61.3	2715	54.9	1571	31.8	422	8.5	1027	20.8
Doctor-diagnosed anxiety (ever)														
No	9695	72.2	3030	31.3	4463	46.0	4414	45.5	1706	17.6	363	3.7	1400	14.4
Yes	3740	27.8	2369	63.3	2257	60.3	1946	52.0	1376	36.8	363	9.7	692	18.5
Conditions for which received counseling ^a^														
Depression	6421	47.8	3944	61.4	3892	60.6	3383	52.7	2037	31.7	497	7.7	1199	18.7
PTSD	4768	35.5	2751	57.7	3020	63.3	2704	56.7	1490	31.3	409	8.6	1005	21.1
Anxiety disorder	3990	29.7	2506	62.8	2447	61.3	2139	53.6	1463	36.7	392	9.8	755	18.9
Other mental health problem	3773	28.1	1848	49.0	2215	58.7	2136	56.6	1153	30.6	390	10.3	771	20.4
Alcohol/drug problems	833	6.2	509	61.1	485	58.2	559	67.1	345	41.4	116	13.9	225	27.0
Any ≥2 conditions	5734	42.7	3619	63.1	3607	62.9	3222	56.2	1962	34.2	518	9.0	1170	20.4
None of the above	3095	23.0	329	10.6	899	29.0	1067	34.5	360	11.6	73	2.4	348	11.2

^a^ Respondents could select all that apply; thus, counts sum to greater than the total and percentages sum to greater than 100%. ^b^ Other mental health professional, such as a social worker, counselor, or therapist. ^c^ As measured by a score of ≥44 on PCL-17 on at least one wave (Waves 1–4). ^d^ As measured by a score of ≥10 on PHQ-8 on at least one wave (Waves 3–4).

**Table 3:** Distributions of personal characteristics, WTC exposures, mental health symptomology, and care-seeking determinants among those that sought counseling in the last 12 months by perceived helpfulness of therapy and adjusted risk ratios (aRR) and 95% confidence intervals (CI).

	Total (*N* = 5429)		Very (*N* = 2192)		aRR ^a^	95% CI		Somewhat (*N* = 2029)		Slightly (*N* = 883)		Not at all (*N* = 217)	
	*N*	%	*N*	% ^b^				*N*	% ^b^	*N*	% ^b^	*N*	% ^b^
Sex												
Men	2934	54.0	1045	36.3	1.00	Reference		1165	40.5	535	18.6	132	4.6
Women	2495	46.0	1147	46.9	1.29	1.18	1.40	864	35.4	348	14.2	85	3.5
Race												
White	4073	75.0	1657	41.3	1.00	Reference		1549	38.6	650	16.2	157	3.9
Black	379	7.0	172	47.1	1.25	1.09	1.43	124	34.0	50	13.7	19	5.2
Hispanic	586	10.8	241	42.6	1.05	0.91	1.20	199	35.2	101	17.8	25	4.4
Asian	170	3.1	51	31.5	0.88	0.64	1.19	67	41.4	35	21.6	9	5.6
Other race	221	4.1	71	33.0	0.72	0.53	0.98	90	41.9	47	21.9	7	3.3
Education at Wave 4												
≤High school/GED	690	12.8	231	34.7	0.91	0.77	1.09	249	37.4	130	19.5	55	8.3
Some college	1428	26.6	513	36.9	0.95	0.85	1.05	538	38.6	271	19.5	70	5.0
College	1594	29.6	671	42.6	0.96	0.88	1.04	598	38.0	254	16.1	51	3.2
Graduate degree	1667	31.0	757	46.1	1.00	Reference		623	38.0	224	13.7	37	2.3
Income at Wave 4												
<$50,000	1360	26.2	517	39.2	1.11	0.98	1.25	481	36.5	241	18.3	80	6.1
≥$50,000 to <$150,000	2499	48.1	1016	41.3	1.04	0.95	1.14	914	37.2	430	17.5	98	4.0
≥$150,000	1340	25.8	569	43.1	1.00	Reference		547	41.4	172	13.0	32	2.4
Age at Wave 4 (years)												
18–44	1175	21.6	484	41.7	0.82	0.73	0.93	435	37.4	199	17.1	44	3.8
45–64	3366	62.0	1325	40.1	0.86	0.79	0.94	1281	38.8	563	17.0	136	4.1
≥65	888	16.4	383	44.8	1.00	Reference		313	36.7	121	14.2	37	4.3
Eligibility group												
Rescue/recovery worker	2429	44.7	944	39.8	1.08	0.98	1.18	904	38.1	413	17.4	112	4.7
Lower Manhattan resident	948	17.5	411	44.1	1.03	0.93	1.14	343	36.8	143	15.4	34	3.7
Lower Manhattan area worker/passerby	2052	37.8	837	41.5	1.00	Reference		782	38.8	327	16.2	71	3.5
WTC exposure score												
None/low	2096	38.6	861	42.2	1.00	Reference		770	37.7	322	15.8	89	4.4
Medium	1867	34.4	765	41.7	1.05	0.96	1.14	687	37.5	320	17.5	61	3.3
High	1013	18.7	365	36.6	0.96	0.86	1.08	407	40.8	179	17.9	47	4.7
Very high	453	8.3	201	44.9	1.21	1.07	1.36	165	36.8	62	13.8	20	4.5
Persistent PTSD ^c^												
Never PTSD	1824	51.1	882	49.0	1.00	Reference		658	36.5	222	12.3	39	2.2
Non-persistent ^d^	1295	36.3	454	35.7	0.76	0.68	0.83	518	40.7	245	19.2	56	4.4
Yes	450	12.6	159	35.7	0.68	0.58	0.81	165	37.1	97	21.8	24	5.4
Conditions for which received counseling in the last 12 months ^e^												
Depression	3075	56.6	1177	38.8	0.85	0.75	0.97	1215	40.0	526	17.3	119	3.9
PTSD	1922	35.4	779	41.1	1.11	1.00	1.24	704	37.2	339	17.9	73	3.9
Anxiety disorder	2109	38.8	812	39.0	0.97	0.87	1.08	839	40.3	357	17.1	74	3.6
Other mental health problem	1937	35.7	750	39.1	0.87	0.79	0.97	750	39.1	332	17.3	86	4.5
Alcohol/drug problems	327	6.0	119	36.6	0.93	0.77	1.12	119	36.6	69	21.2	18	5.5
Any ≥2 conditions	2775	51.1	1068	39.0	1.11	0.96	1.28	1092	39.8	480	17.5	101	3.7
None of the above	776	14.3	323	44.8	0.94	0.82	1.08	238	33.0	121	16.8	39	5.4
Counseling frequency in last 12 months												
>1 per week	299	5.7	148	50.7	1.57	1.34	1.85	99	33.9	40	13.7	5	1.7
1 per week	1768	33.4	797	45.4	1.25	1.11	1.41	649	37.0	267	15.2	42	2.4
2–3 times per month	1274	24.1	539	42.8	1.23	1.09	1.39	515	40.9	176	14.0	30	2.4
1 per month	880	16.6	331	38.0	1.12	0.97	1.29	355	40.7	148	17.0	38	4.4
<1 per month	1067	20.2	348	33.0	1.00	Reference			36.3	235	22.3	90	8.5
Received medication for a mental health problem in the last 12 months												
No	2335	43.0	1011	44.4	1.00	Reference		829	36.4	347	15.2	92	4.0
Yes	3094	57.0	1181	38.8	0.91	0.83	0.99	1200	39.4	536	17.6	125	4.1
Sought counseling prior to the last 12 months												
No	543	10.6	175	32.8	1.00	Reference		209	39.2	111	20.8	38	7.1
Yes	4601	89.4	1937	42.7	1.34	1.14	1.57	1718	37.9	717	15.8	162	3.6
Ever been without insurance in last 12 months												
No	4963	92.1	2018	41.5	1.00	Reference		1862	38.2	794	16.3	194	4.0
Yes	425	7.9	159	38.2	1.17	0.99	1.38	150	36.1	85	20.4	22	5.3
Unmet mental healthcare need in last 12 months												
No	4982	94.2	2090	42.7	1.00	Reference		1899	38.8	755	15.4	147	3.0
Yes	306	5.8	50	16.9	0.45	0.31	0.65	82	27.8	100	33.9	63	21.4
Previous unmet mental healthcare need ^f^												
No	2935	68.9	1309	45.3	1.00	Reference		1082	37.4	416	14.4	84	2.9
Yes	1327	31.1	445	34.2	0.84	0.76	0.93	510	39.2	276	21.2	71	5.5

^a^ Risk ratio represents the relative probability that perceived helpfulness = very helpful vs. all other categories (somewhat, slightly, and not at all). ^b^ Denominators exclude *N* = 108 missing responses for perceived helpfulness of therapy in last 12 months. ^c^ Evaluated among those not missing any PCL items across four Waves (*N* = 3569); persistent PTSD as measured by a score of ≥44 at all waves. ^d^ Non-persistent PTSD was defined as those who had PCL-17 scores ≥44 on at least one Wave (1–4), but not at all four Waves. ^e^ Respondents could select all that apply; thus, counts sum to greater than the total and percentages sum to greater than 100%. Each risk ratio represents the comparison between those who received counseling for each condition vs. not that condition (i.e., all other indications). ^f^ As assessed at Waves 2 and 3.
